# Acute Myopericarditis as the First Manifestation of Familial Mediterranean Fever: A Case Report

**DOI:** 10.7759/cureus.54170

**Published:** 2024-02-14

**Authors:** Abdalla Khalil, Andrew Greenhalgh, Shovhit Gurung, Harmeet Chana

**Affiliations:** 1 Acute Medicine, Northwick Park Hospital, London North West University Healthcare NHS Trust, London, GBR; 2 Radiology, Nortwick Park Hospital, London North West University Healthcare NHS Trust, London, GBR

**Keywords:** polyserositis, pericardial effusion, acute myopericarditis, auto-inflammatory disorders, familial mediterranean fever

## Abstract

Familial Mediterranean fever (FMF) is an autoinflammatory disorder, characterized by recurrent episodes of fever and polyserositis, and usually presents during the first two decades of life. Acute pericarditis is a rare manifestation of FMF and typically presents with other symptoms of the inflammatory disorder. A 27-year-old Arabian male presented to our hospital with pleuritic chest pain and shortness of breath while lying flat. His electrocardiogram showed changes suggestive of pericarditis, and his inflammatory markers and troponin were raised. His echocardiogram revealed a moderate-sized pericardial effusion with septa and a normal left ventricular function. He had a strong family history of FMF and consanguinity of the parents. He was treated for acute myopericarditis with colchicine and ibuprofen, and his symptoms improved gradually along with his inflammatory markers and troponin. Six weeks after discharge, he had a cardiac MRI, which revealed a thickened pericardium with profound enhancement (features suggestive of pericarditis) and no signs of myocarditis. He was asymptomatic, and his markers and troponin were within the normal range. His colchicine medication was continued indefinitely, and he was referred to a tertiary care hospital with a specialized periodic fever clinic for follow-up and genotype testing.

## Introduction

Familial Mediterranean fever (FMF) is a hereditary auto-inflammatory disease characterized by periodic episodes of fever and serositis (mainly peritonitis, pleuritis, and arthritis) [1]. It is common among Turks, Arabs, Armenians, and non-Ashkenazi Jews who have a high rate of *MEFV* gene mutation at the short arm of chromosome 16 [2].

The pyrin protein is an important regulator of inflammation and immune response, especially in serosa (e.g., peritoneum, pleura, synovial membrane, and pericardium). In patients with FMF, the *MEFV* gene, which is responsible for the production of pyrin, is mutated and causes an inappropriate inflammatory response manifested by the clinical features of FMF [2].

The diagnosis of FMF generally takes place during the first decade of life (65% of the patients within the first 10 years of life and 90% within the first 20 years). Late-onset FMF is less common and described in patients with milder forms [3,4]. In a huge multicentre study in Turkey (2,838 patients), clinical features of FMF included peritonitis (93.7%), fever (92.5%), arthritis (47.4%), pleuritis (31.2%), myalgia (39.6%), and erysipelas-like erythema (20.9%) [5]. The criteria developed at the Tel Hashomer Medical Centre remain the most widely used and well-accepted criteria. Several other diagnostic criteria have also been proposed, like the Livneh criteria, which had a sensitivity of 95% and specificity of 97% [6] (Table 3).

## Case presentation

A 27-year-old Arabian male, who was normally fit and well and had no past medical history, presented to a local hospital with pleuritic chest pain, described as a stabbing sensation radiating to both shoulders. His troponin and D-dimer were both negative, and his inflammatory markers were raised. As such, he was treated for presumed pericarditis with colchicine and ibuprofen, and he discharged himself against medical advice the next day.

However, despite being compliant with his treatment, the patient presented to our hospital (two days after discharge) with worsening sharp pleuritic chest pain radiating to the left shoulder. The pain was worse when lying flat and better when sitting up. The patient denied any shortness of breath, palpitations, or syncope. The patient did feel dizzy on occasion but had no heart failure symptoms.

The patient had never experienced chest pain symptoms like this in the past, and there was no history of recent viral illnesses, diarrhea or vomiting, any weight loss, or night sweats. The patient had not traveled in the last year and had no contact with any unwell person or anyone with tuberculosis. He did not drink alcohol and did not use recreational drugs apart from occasionally smoking shisha.

The patient had no family history of cardiac diseases or autoimmune conditions but did have a first-degree relative (brother is known to have FMF with episodic chest pain currently on colchicine), and consanguinity of his parents.

**Figure 1:**
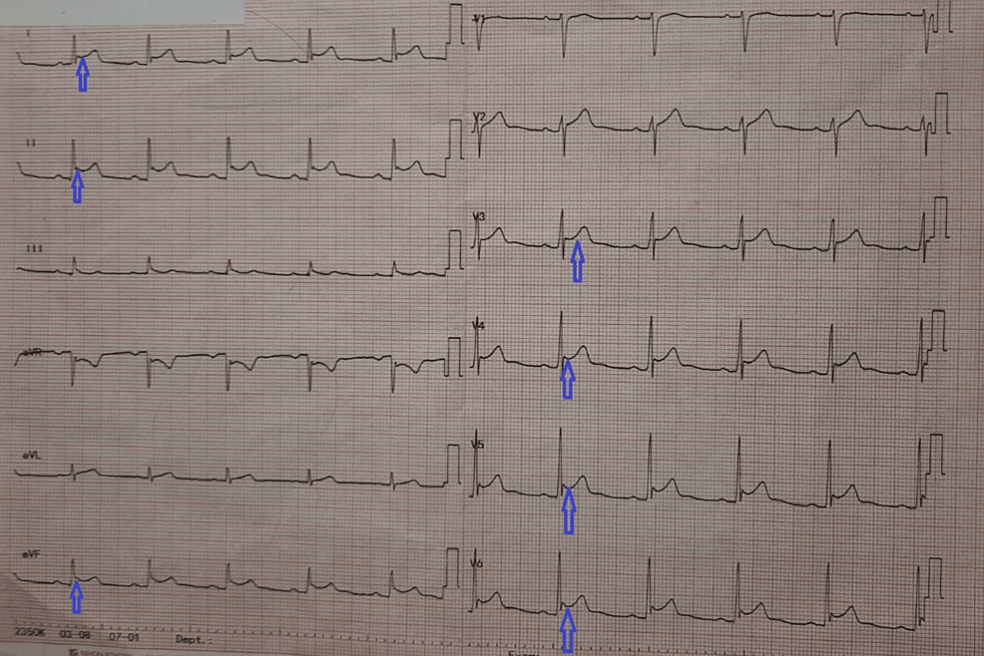
12 Leads electrocardiogram. The figure shows widespread saddle ST elevations suggestive of acute pericarditis.

The examination was largely unremarkable. He was warm and well-perfused, on auscultation his lungs were clear, and he had normal heart sounds with no pericardial or pleural rub. The abdominal examination was normal, and his calves were soft and non-tender. There were no signs of heart failure, no other stigmata of systemic disease, lymphadenopathy, or pharyngitis. On investigation, the patient had widespread saddle ST elevations on his electrocardiogram (Figure 1).

The patient had a rounded cardiac shadow on a chest X-ray with clear lung fields. Inflammatory markers were raised with a C-reactive protein of 202 mg/L at its highest and a raised troponin of 947 ng/L but a negative BNP (B-type natriuretic peptide) of 70 pg/mL. His autoimmune, vasculitis, extended viral, and bacterial screens were negative, along with blood, urine, and throat cultures. Metabolic tests for thyroid dysfunction and diabetes were negative, and streptolysin antibody and tuberculosis QuantiFERON assays were also negative (Tables 1, 2).

**Table 1:** Autoimmune and infective screen. ANA: antinuclear antibody, ANCA: anti-neutrophil cytoplasmic antibodies, HSV: herpes simplex virus

Investigations	Result	References
ANA pattern level	Negative	
Complement c3 level, blood	1.38	0.9–1.8 g/L
Complement c4 level, blood	0.40	0.1–0.4 g/L
dsDNA ab level, blood	0.7	0–10
ANCA immunofluorescence, blood	Negative	
Smoot muscle antibody pattern, blood	Negative	
Tuberculosis QuantiFERON assay, blood	–0.01 Negative	
Mitochondrial ab level, blood	Negative	
Rheumatoid factor, blood	14.2	0–30
HIV-1 and HIV-2 serology, blood	Non-reactive	
Hepatitis B virus surface ag, blood	Non-reactive	
Hepatitis C virus ab, blood	Non-reactive	
Parvovirus B19 IgM, blood	<0.10	
Anti streptolysin O titer, blood	400	200–400 IU/mL
Chlamydia and gonorrhoea, urine	Negative	
HSV IgM	Negative	

**Table 2:** Inflammatory markers and troponin levels during the patient's admission and after discharge. WBC: white blood cell, CRP: C-reactive protein, ESR: erythrocyte sedimentation rate.

Investigation	Day 1	Day 3	Day 4	Day 5	Day 7	Day 33	Reference
WBC	17.000	-	-	-	7.700		3-10 x 10^9^/L
Troponin	<5	947	751	236	32	6	0-14 ng/L
CRP	7.5	166	202	151	114	6.5	0.0-5.0 mg/L
ESR	37-45					10-20	10 mm/hr

**Figure 2:**
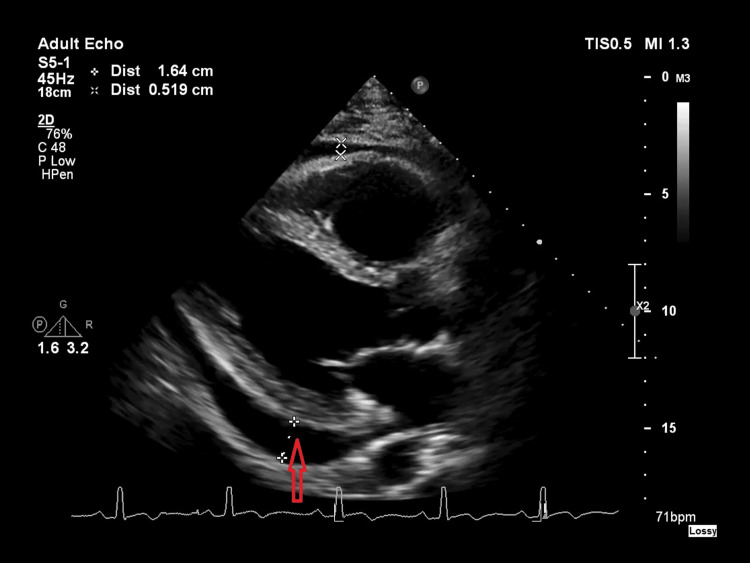
Echocardiography parasternal long axis view. The figure shows moderate circumferential pericardial effusion, with a maximum diameter of 1.6 cm posterior to the left ventricle.

**Figure 3:**
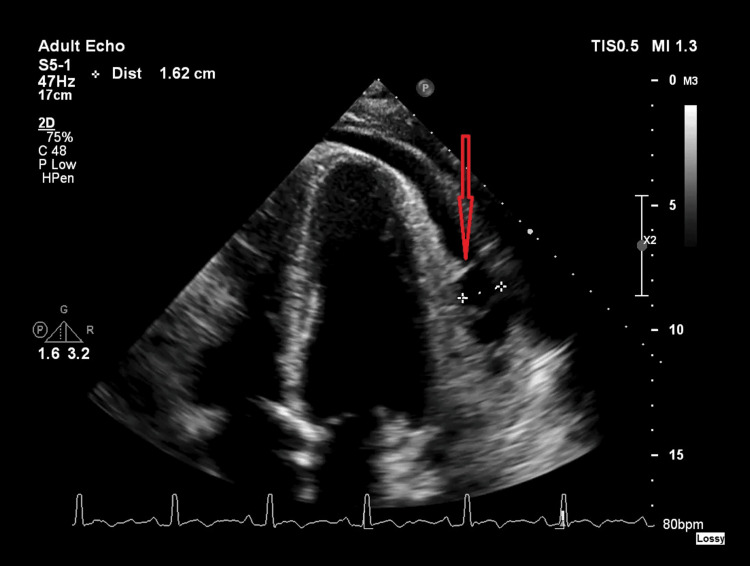
Echocardiography apical view. The figure reveals the pericardial effusion with a septa posterior to the left ventricle.

The patient underwent an inpatient echocardiogram, which showed a normal ventricular function and no regional wall motion abnormalities or valve pathology. There was a moderate circumferential pericardial effusion, with a maximum diameter of 1.6 cm posterior to the left ventricle and fibrous septa present, suggesting a degree of chronicity (Figures 2, 3).

There were no signs of regional wall motion abnormalities, indicating a myopericarditis picture, that is, a raised troponin but normal heart function. The patient continued his treatment with NSAIDs and colchicine, and his symptoms improved during this admission, with his troponin dropping to 32 ng/L and his C-reactive protein dropping to 114 mg/L.

**Figure 4:**
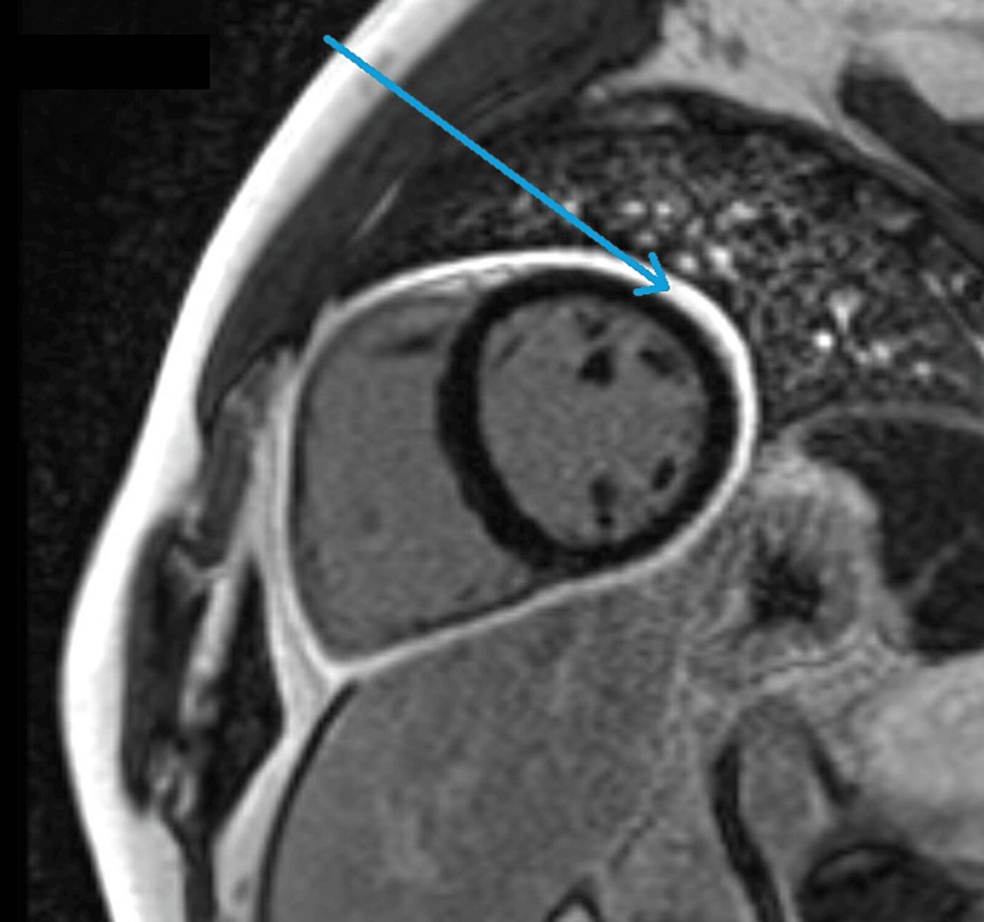
MRI cardiology 2 chamber short axis. Acquired 10 minutes after administration of 15 mL IV Gadovist using an inversion time of 300 ms, which showed a normal appearance of both LV and RV and a very bright circumferential white rim around the ventricles representing pericardial thickening with enhancement (acute pericarditis).

**Figure 5:**
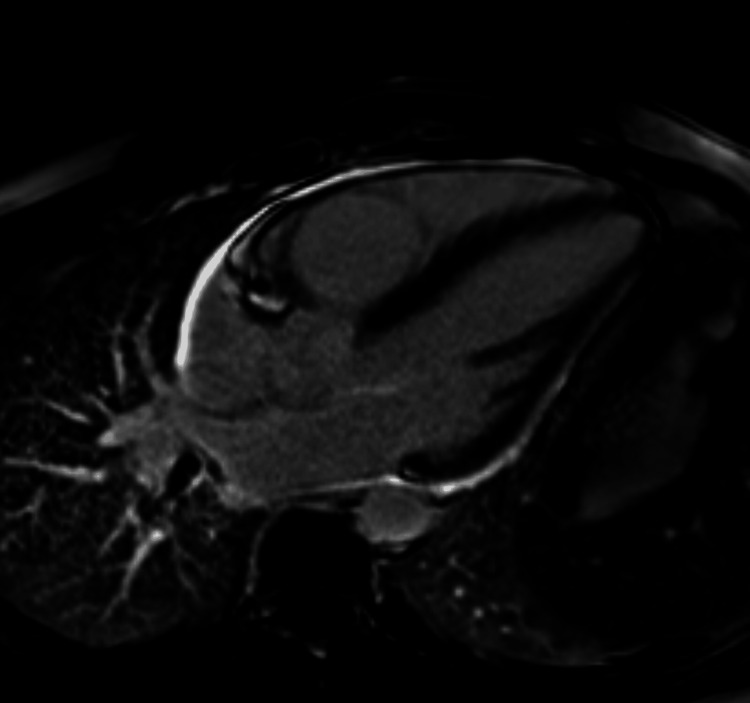
MRI cardiology late gadolinium 3 chambers image. Acquired 10 minutes after administration of IV Gadovist using an inversion time of 300 ms, revealing the normal appearance of LV and RV and no CMR features of myocarditis.

Six weeks after discharge, the patient had a cardiac magnetic resonance scan (MRC) which showed normal appearances of the left and right ventricles. A bright circumferential white rim around the ventricles shows the pericardial thickening with enhancement (10 minutes after IV Gadolinium). Features were suggestive of pericarditis with no evidence of acute myocarditis (Figures 4, 5).

The patient was asymptomatic, and all his inflammatory markers and troponin were within normal levels. His white blood cell and differential count normal were also normal. This patient meets the criteria for FMF (Table 3).

**Table 3:** Criteria for diagnosis of FMF Adapted with permission from Livneh et al. [6] FMF: familial Mediterranean fever.

A	Major criteria
	Typical attacks
1	Peritonitis (generalized)
2	Pleuritis (unilateral) or pericarditis
3	Monoarthritis (hip, knee, ankle)
4	Fever alone
	Typical attack recurrent ≥ 3, fever of 38 °C or > rectal, and short (between 12 hours and 3 days)
B	Minor criteria
	Incomplete attacks
1	Abdomen
2	Chest
3	Joint
4	Exertional leg pain
5	Favorable response to colchicine
C	Supportive criteria
1	Family history of FMF
2	Appropriate ethnic origin
3	Age less than 20 years at the time of diagnosis
4	Severe requiring bed rest
5	Spontaneous remission
6	Symptoms free intervals
7	Transient inflammatory response (WBCs, ESR, fibrinogen, or serum amyloid A)
8	Episodic hematuria or proteinuria
9	Negative laparotomy or appendectomy
10	Consanguinity of the parents
D	≥1 Major criteria, ≥2 minor criteria, 1 minor plus 5 supportive criteria

The patient will continue colchicine indefinitely to suppress chronic inflammation and reduce the risk of amyloidosis. He was referred to a tertiary care hospital with a periodic fevers clinic for a specialized follow-up and genotype check. 

## Discussion

The prevalence of pericarditis in patients with FMF varies from 0.7% to 1.4% in some studies to 3.6% in one prospective study that used echocardiography for diagnosis [7, 8]. Pericarditis typically presents simultaneously with other FMF symptoms and has been reported rarely as the only manifestation of the FMF attack [9, 10].

In a study in pediatrics, pericarditis was diagnosed in about 11% of children with FMF presenting with chest pain and showed that pericarditis was especially seen in those with M694V and E148Q mutations of MEFV [11]. A rare complication of pericardial disease, constrictive pericarditis, was reported in a patient with FMF and protein-losing enteropathy which resolved completely after six months of regular colchicine therapy [12].

Cardiac tamponade as a rare manifestation of FMF was also reported with a massive pleural and pericardial effusion [13]. The goals of therapy for FMF are to prevent acute attacks and minimize subclinical inflammation between attacks, and to prevent the development and progression of amyloidosis. Colchicine remains the first line of treatment for FMF, which was approved by the US Food and Drug Administration (FDA) in 2009 and is only for patients above four years old. It should be started as soon as a clinical diagnosis is established and should be continued indefinitely [14].

Colchicine demonstrated efficacy in preventing acute inflammatory episodes as well as preventing or slowing the progression toward amyloidosis [15]. Canakinumab is the only FDA-approved cytokine blocker for the treatment of colchicine-resistant FMF in the United States and was found to be effective and tolerable [16].

Our patient presented to the hospital with acute myopericarditis with high inflammatory markers and a raised troponin. His ECG and echocardiography confirmed the diagnosis. He had a strong family history of FMF (brother), the proper ethnicity, and a history of consanguinity with the parents. His chest pain gradually responded to colchicine, and his inflammatory markers and troponin trended down with medication and rest. His list of investigations for other causes of myopericarditis was negative.

Our patient had two minor criteria for diagnosis of FMF and four supportive criteria, and he will be followed by the periodic fever clinic and amyloidosis section in a tertiary care hospital.

## Conclusions

Acute myopericarditis is a rare manifestation of FMF, which is unlikely to occur without other FMF findings and rarely after the first two decades. An Arabian male patient in his third decade presented with isolated acute myopericarditis as his first manifestation of FMF. He responded clinically, and his inflammatory markers and troponin gradually improved while on colchicine therapy.

He met the criteria for diagnosis of FMF, and his investigations for other causes of acute myopericarditis were negative. Both his echocardiography and cardiac MRI confirmed the presence of pericarditis with normal left ventricular function and resolution of the pericardial effusion after colchicine therapy, and he will be reviewed in a periodic fever clinic at a tertiary care hospital.

## References

[1] Onen F (2006). Familial Mediterranean fever. Rheumatol Int.

[2] Rigante D, Cantarini L, Imazio M (2011). Autoinflammatory diseases and cardiovascular manifestations. Ann Med.

[3] Demirkaya E, Saglam C, Turker T (2016). Performance of different diagnostic criteria for familial Mediterranean fever in children with periodic fevers: results from a multicenter international registry. J Rheumatol.

[4] Özdel S, Özçakar ZB, Kunt SŞ, Elhan AH, Yalçınkaya F (2016). Late-onset disease is associated with a mild phenotype in children with familial Mediterranean fever. Clin Rheumatol.

[5] (2005). Familial Mediterranean fever (FMF) in Turkey: results of a nationwide multicenter study. Medicine (Baltimore).

[6] Livneh A, Langevitz P, Zemer D (1997). Criteria for the diagnosis of familial Mediterranean fever. Arthritis Rheum.

[7] Tutar HE, Imamoglu A, Kendirli T, Akar E, Atalay S, Akar N (2001). Isolated recurrent pericarditis in a patient with familial Mediterranean fever. Eur J Pediatr.

[8] Tutar E, Yalçinkaya F, Ozkaya N, Ekim M, Atalay S (2003). Incidence of pericardial effusion during attacks of familial Mediterranean fever. Heart.

[9] Kees S, Langevitz P, Zemer D, Padeh S, Pras M, Livneh A (1997). Attacks of pericarditis as a manifestation of familial Mediterranean fever (FMF). QJM.

[10] Yoshioka K, Furumitsu Y, Sano T, Miyamoto T, Agematsu K (2014). Acute pericarditis as the first manifestation of familial Mediterranean fever: a possible relationship with idiopathic recurrent pericarditis. Intern Med.

[11] Kilic A, Varkal MA, Durmus MS (2015). Relationship between clinical findings and genetic mutations in patients with familial Mediterranean fever. Pediatr Rheumatol Online J.

[12] Gökçe I, Gökçe S, Kılıç A, Bozlar U, Kocaoğlu M, Ongürü O, Gök F (2011). Familial Mediteranean fever with protein-losing enteropathy due to constrictive pericarditis. World J Pediatr.

[13] Malek A, Zeraati T, Sadr-Nabavi A, Vakili N, Abbaszadegan MR (2022). Cardiac tamponade: a rare manifestation of familial Mediterranean fever. Case Rep Rheumatol.

[14] Kallinich T, Haffner D, Niehues T (2007). Colchicine use in children and adolescents with familial Mediterranean fever: literature review and consensus statement. Pediatrics.

[15] Leung YY, Yao Hui LL, Kraus VB (2015). Colchicine - update on mechanisms of action and therapeutic uses. Semin Arthritis Rheum.

[16] Karabulut Y, Gezer HH, Duruöz MT (2022). Canakinumab is effective in patients with familial Mediterranean fever resistant and intolerant to the colchicine and/or anakinra treatment. Rheumatol Int.

